# Preliminary Evidence of Efficacy, Safety, and Treatment Satisfaction with Tirbanibulin 1% Ointment: A Clinical Perspective on Actinic Keratoses

**DOI:** 10.3390/ph16121686

**Published:** 2023-12-04

**Authors:** Elena Campione, Antonia Rivieccio, Ruslana Gaeta Shumak, Gaetana Costanza, Terenzio Cosio, Sara Lambiase, Virginia Garofalo, Fabio Artosi, Flavia Lozzi, Claudia Freni, Alice Romeo, Emi Dika, Mattia Falconi, Luca Bianchi

**Affiliations:** 1 Dermatology Unit, Department of Systems Medicine, University of Rome Tor Vergata, 00133 Rome, Italy; anthis94@hotmail.it (A.R.); ruslanagaetashumak@gmail.com (R.G.S.); terenziocosio@gmail.com (T.C.); fabio.artosi994@gmail.com (F.A.); flavia.lozzi@alumni.uniroma2.it (F.L.); l.biamchi@uniroma2.it (L.B.); 2 Department of Experimental Medicine, University of Rome Tor Vergata, 00133 Rome, Italy; 3 Department of Biology, University of Rome Tor Vergata, Via della Ricerca Scientifica, 00133 Rome, Italy; claudia.freni@uniroma2.it (C.F.); alice.romeo@uniroma2.it (A.R.); falconi@uniroma2.it (M.F.); 4 Oncologic Dermatology Unit, IRCCS Azienda Ospedaliero-Universitaria di Bologna, DIMEC, University of Bologna, 40126 Bologna, Italy; emi.dika3@unibo.it

**Keywords:** tirbanibulin, actinic keratosis, field of cancerization

## Abstract

Background: Actinic keratosis is a common precancerous skin lesion that can progress into invasive squamous cell carcinomas. Many topical treatments for actinic keratoses often have poor tolerability and prolonged duration. Tirbanibulin is a novel synthetic drug with potent antitumor and antiproliferative activities. Methods: We conducted a single-center, prospective and observational study using tirbanibulin ointment on a 25 cm^2^ area for 5 consecutive days on 30 participants with AKs on the face or scalp. They were followed for at least 57 days to assess the safety profile and efficacy of the drug as well as treatment satisfaction. We evaluated six signs of local skin reaction (LSR): erythema, scaling, crusting, swelling, blisters/pustules, and erosions/ulcerations, grading the severity as mild, moderate, or severe. The effectiveness was evaluated both clinically and dermoscopically. The treatment satisfaction was assessed using the Treatment Satisfaction Questionnaire for Medication (TSQM 1.4). Results: On day 57, 70% of the patients showed a complete clinical and dermoscopic response. The highest scores obtained from the TSQM 1.4 were more evident in the convenience and side effects domains. Most LSRs, including erythema (83.3%), scaling (30%), and swelling (3.3%), occurred on day 8 but resolved spontaneously. Conclusion: Tirbanibulin is a viable therapeutic option with a short regimen treatment and good tolerability, which favors therapy adherence.

## 1. Introduction

Actinic keratosis (AK) is an intraepithelial proliferation of atypical keratinocytes that can progress into invasive squamous cell carcinomas [1]. Ultraviolet radiation (UVR) is the most important risk factor for the development of this kind of lesion. Cumulative exposure leads to genetic alterations, which at first induce subclinical atypia and then progress to keratinocyte neoplasms.

One of the most common UVR-related mutations concerns tumor protein 53 gene (TP53) encoding p53, a tumor suppressor essential in DNA stability control and cell cycle arrest after DNA damage. TP53 mutations have been found in cutaneous squamocellular carcinoma (cSCC), AK and sun-damaged skin but also in normal sun-exposed skin [2], suggesting that this mutation appears in the early development of keratinocyte carcinomas. Many authors consider AK to be an in situ SCC that may progress to the invasive stage [3]. AKs usually arise on fair skin, mainly in elderly patients with a long history of chronic sun exposure [4]. A correct diagnosis and treatment choice are crucial due to the possible progression of AK into cutaneous squamous cell carcinoma (cSCC) [4,5,6]. The rates of progression into invasive SCC have been estimated at 0–0.075% per AK per year for individuals without a previous history of non-melanoma skin cancer (NMSC) and 0–5% for those with a history of NMSCs [5,6]. Generally, AKs are precancerous skin lesions. Thus, diagnosis in the early stages allows a conservative approach and a higher cure rate for the disease. Its incidence is equal to 10% in subjects with light skin and under the age of 30, while it exceeds 80% after the age of 60 [7]. The main risk factors are light phototype, advanced age, sunburns during childhood, inadequate photo exposure, and immunosuppression. From a clinical point of view, Aks present as erythematous-squamous macules, the color of which can vary from brownish yellow to whitish. On palpation, a wrinkled consistency is noticed, often defined as “sandy” by patients, which represents the most characteristic diagnostic feature. At dermoscopy, the diagnosis is based on the search for specific patterns: erythema forming a pink-reddish vascular pseudo-network surrounding hair follicles, yellowish-white scales, thin and wavy vessels surrounding the follicles, and follicular openings filled with keratotic plugs [8]. The latter are also used to classify Aks into three degrees of severity [9]. AK dermoscopy is characterized by the presence of specific diagnostic criteria, and three different clinical grades of AKs, according to Olsen, correspond dermoscopically to three different models [10].

AKs are mainly localized on the areas that are most photo-exposed: face, scalp in bald subjects, auricles, back of the hands, and décolleté. They can be isolated lesions, more often in young subjects, or multiple, especially in the elderly where they are often localized within a larger and photo-damaged area of the skin called the “cancerization field”(FC). AKs may spontaneously regress, remain unchanged, or progress to SCC. Since the individual risk of degeneration is unpredictable, it is common consensus in clinical practice to treat all AKs [11]. The treatment of the FC depends on the presence of some risk/progression factors such as the number of lesions, the presence of severe skin photodamage and/or immunosuppression and the positive history of other NMSC. Currently, clinicians apply useful non-invasive imaging techniques such as dermoscopy and reflectance confocal microscopy (RCM), which are useful not only in diagnosing but also in monitoring the effect of treatments over time [12]. The therapy of AKs can be divided into a preventive therapy, aimed at reducing the risk of its appearance, and a curative one, aimed at treating the already formed AKs and possibly the surrounding FC [13,14]. Regarding curative treatments, current scientific evidence strongly supports cryotherapy, mainly for single lesions, and topical therapy with 5-Fluorouracil (5-FU) or imiquimod [15]. More conflicting, in cost/benefit terms, are instead the data regarding topical diclofenac and photodynamic therapy (PDT) [16]. The simplest and most used method to treat AKs is represented by cryotherapy, which has reported a success rate between 57 and 98.8%; discomfort/pain during the procedure and post-treatment discoloration are the main side effects but they can be minimized by shorter freezing times. Treatment with 0.5% 5-FU cream alone or with the addition of 10% salicylic acid has shown an efficacy rate of approximately 75% [17]. The main side effect, as well as the most frequent cause of discontinuation, was local irritation. At week 4, a complete clearance of 50% of the treated lesions and a partial clearance of 28% has been noticed [18]. Imiquimod cream in a 3.75% or 5% formulation guarantees excellent results but the treatment could be associated with discomfort for side effects such as skin irritation, erythematous-crusted lesions, and sometimes flu-like symptoms [19]. Even the topical medical device piroxicam 0.8% with sunscreen filters favors a regression of grade I and II AKs with good outcomes. Its use could be considered to treat FC, as documented in numerous clinical real-life studies and also in transplant patients or in those taking photosensitizers antihypertensives drugs [20,21,22,23,24]. Preventive therapy of AKs, on the other hand, is mainly based on photo sunscreen [25]. Oral nicotinamide (a derivative of vitamin B3) at a dosage of 1 g/daily is a valid chemopreventive treatment in patients with almost 2 or more NMSCs in their medical history [26,27]. The mechanism of action of nicotinamide is the activation of DNA repair enzymes through PARP1. Recently, topical tirbanibulin 1% ointment—an inhibitor of mitotic spindle formation—was introduced as a therapeutic strategy. Tirbanibulin disrupts microtubules, via direct binding to tubulin, inducing cell cycle arrest and apoptotic death of proliferating cells, and is associated with disruption of Src tyrosine kinase signaling [28]. Two randomized, double-blind, parallel-group, vehicle-controlled, phase 3 clinical trials were conducted in 702 subjects with 4–8 non-hyperkeratotic, non-hypertrophic AKs within a contiguous treatment area of 25 cm^2^ on the face or scalp [29]. On each scheduled dosing day, the ointment was applied to the entire treatment area. In the tirbanibulin group, 96% of patients had skin type I, II, or III according to the Fitzpatrick classification. Efficacy, measured as complete (primary endpoint) and partial clearance rate, was assessed on day 57 [29]. Pooled results from both studies demonstrated complete clearance in 49% of patients (n = 174) and partial clearance in 72% of patients (n = 255) treated with tirbanibulin [29].

Here we report our real-life clinical experience in 30 patients affected by grade I AKs, located on the face and scalp, treated with tirbanibilin 1% ointment for 5 consecutive days. Many topical treatments for actinic keratoses often have poor tolerability and prolonged duration. This leads to reduced patient adherence and compromises the success of the treatment. Tirbanibulin exhibits potent antitumor and antiproliferative activity. Its antiproliferative activity is attributed to its ability to reversibly bind to tubulin, inhibiting its polymerization and promoting microtubule breakdown, thereby inducing cell cycle arrest and apoptosis in proliferating cells. Unlike other treatments for actinic keratoses, tirbanibulin does not induce tissue necrosis, coupled with a low rate of skin inflammation not resulting in severe local skin reactions. The topical application of tirbanibulin is characterized by good tolerability and a favorable safety profile. We obtained excellent outcomes in terms of effectiveness, safety, and treatment satisfaction. The short regimen of the tirbanibulin application and the great tolerability increased the patient’s adherence and satisfaction.

## 2. Results

### 2.1. Demographic Characteristics of Enrolled Patients

We enrolled 30 patients; 18/30 patients were male and 12/30 were females, respectively, with an average age of 75 (range 60–93 years old). At baseline, demographic characteristics, phototype, chronic sun exposure, previous diagnosis of basal cell carcinoma (BCC), number and location of AKs, previous treatments, presence of comorbidities, and concurrent therapies were recorded for all patients (Table 1). We evaluated the color of the skin and texture of the hair based on Fitzpatrick’s skin phototypes [30]. In total, 40% of our patient population showed Fitzpatrick phototype I, 53.3% type II, and 6.7% type III. The studied population had a history of chronic sun exposure due to occupational or recreational reasons. In total, 46.7% had been treated surgically for previous BCC. Additionally, 90% of patients showed an extensive FC, and we found the presence of 2–8 AKs grade I according to Olsen. In the enrolled population, 4 patients had never undergone any treatment for AKs, while 26 patients received at least one treatment: piroxicam 0.8% and sun filter (27%), cryotherapy (24%), 5-Aminolevulinic acid patch (4%), diclofenac (24%), 5-fluorouracil 0.5% (5-FU) and salicylic acid 10% (8%), 5-fluorouracil 5% (8%) imiquimod 5% (15%), and photodynamic therapy (19%).

**Figure 1:**
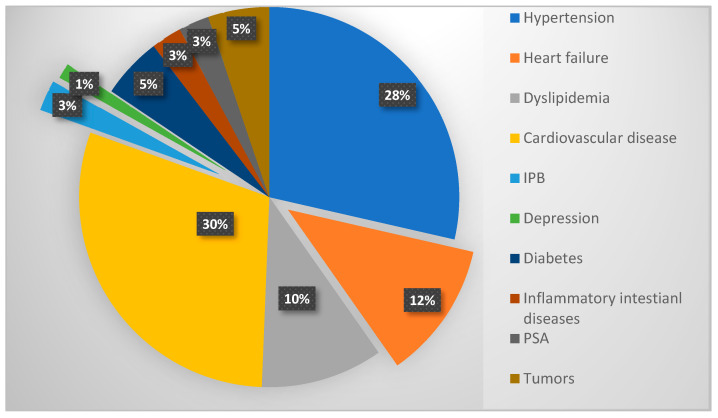
Comorbidities in enrolled patients (*n* = 26).

Out of the 30 patients under investigation, 26 were affected by various comorbidities for which they were undergoing specific systemic therapies. Only four patients from the examined population had no comorbidities and were not under any systemic treatment drugs. Previous medical history of the analyzed population included cardiovascular disease (29.87%), arterial hypertension (2.57%), heart failure (11.68%), dyslipidemia (10.38%), diabetes mellitus type II (5.2%), benign prostatic hyperplasia IPB (2.6%), psoriatic arthritis PsA (2.6%), inflammatory bowel disease (IBL) (2.6%), other tumors (5.2%), and depression (1%) (Figure 1).

Furthermore, the enrolled patients had been under long-term treatment with several systemic medications for the different associated comorbidities as follows: acetylsalicylic acid (20.75%), thiazide diuretics (11.3%), potassium-sparing diuretics (5.66%), angiotensin II receptor blockers (11.3%), statins (11.3%), antiplatelet (9.43%), metformin (7.54%), incretin (1.88%), insulin (1.88%), levotiroxina (7.54%), beta-blockers (3.77%), calcium channel blockers (3.77%), and carbamazepine (1.88%) as reported in Table 2.

The diagnosis of AKs was made clinically and dermoscopically. The lesions were located within a 25 cm^2^ area on the scalp in 53.3% and on the face in 46.7%, and were treated with 1% tirbanibulin ointment for 5 consecutive days. All patients reported having completed all five daily doses of the treatment, and none of them dropped out of the study. The first follow-up was conducted around day 8 after the first tirbanibulin ointment application, followed by assessments at two weeks, one month, and finally at approximately two months.

### 2.2. Safety in Term of LSR

LSRs were observed in 86.67% of the patients (n = 26) while 13.33% (n = 4) did not experience any local adverse events related to the treatment with tirbanibulin 1% ointment. LSRs were observed between 2 and 15 days following the beginning of therapy. As reported from RCTs, day 8 after drug application is considered the LSR peak day. We then assessed the gradual resolution of LSRs on day 15 and 29 in accordance with phase 3 clinical trials [31]. All local reactions had an average resolution duration of 10 days and resolved spontaneously without any lasting effects.

**Figure 2:**
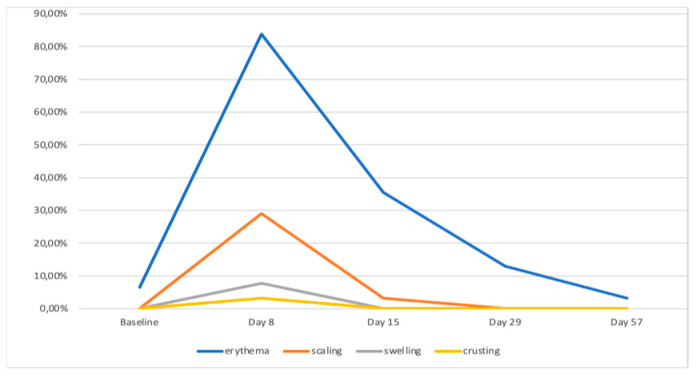
Temporal reduction of LSRs across different follow-up time points.

Considering the duration of the treatment, all patients could experience multiple side effects simultaneously. The most commonly observed was erythema on the application site in 83.3% (n = 25), mild erythema in 30% (n = 9) of cases, and moderate erythema in 53.3% (n = 16) of cases. This local skin effect partially resolved on day 15 of follow-up with a rate of mild erythema persistence in 40% (n = 12) of cases, and completely resolved by day 29 of follow-up. Desquamation was the second most frequent LSR recorded in 30% (n = 9) of cases, with 26.6% (n = 8) presenting the mild form and 3.33% (n = 1) in a moderate form, also resolving completely within day 29 of follow-up. Only one case of crusting formation and two cases of swelling in the treated area were observed. Additionally, the enrolled patients reported itching, burning, and pain at the site of tirbanibulin application, respectively in 53.3% (n = 16), 50% (n = 15), and 10% (n = 3) during the 5 days of the treatment application period, which resolved spontaneously (Figure 2). The LSRs did not cause discomfort to the patients, who all completed the treatment.

All local skin reactions had an average resolution duration of 10 days and all these signs resolved spontaneously without the need for any further medical intervention. No patient experienced systemic effects related to the drug.

Statistical analysis was conducted to compare each clinical and dermoscopic parameter at baseline (BL) with those observed on day 8 (T8), day 15 (T15), day 29 (T29), and day 57 (T57). The resolution of local skin reactions over the monitoring period was statistically significant (Figure 2; ANOVA test; *p* < 0.01).

### 2.3. Efficacy

**Figure 3:**
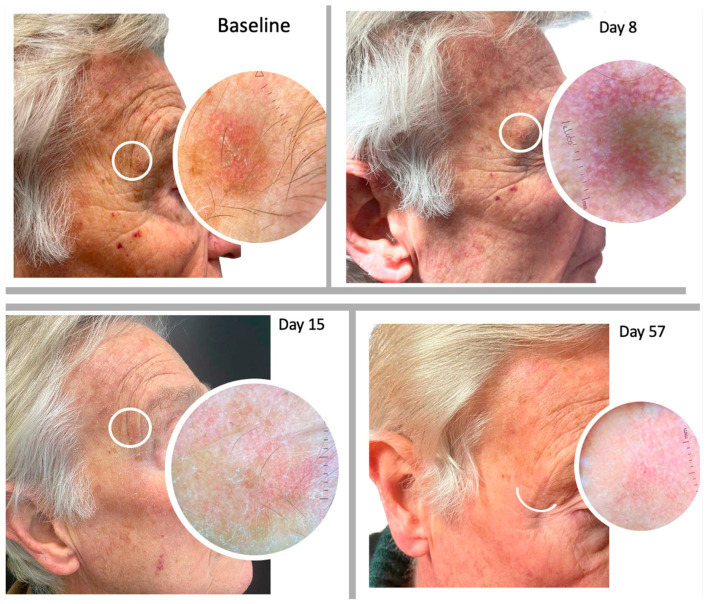
Comparison of clinical manifestations of AKs before and after treatment with 1% tirbanibulin ointment. The images show the presence of flakiness and irritation at the initial stage as well as illustrate the resolution at the end of the treatment.

**Figure 4:**
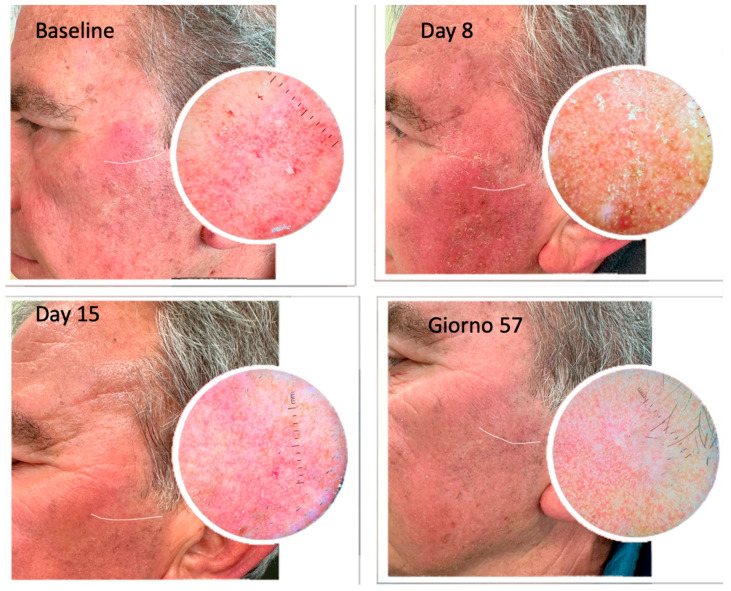
AKs before and after treatment with 1% tirbanibulin ointment.

**Figure 5:**
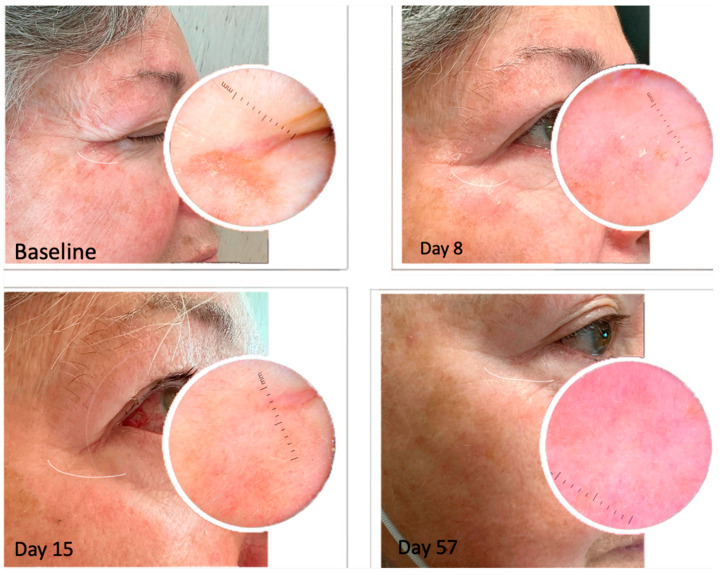
Comparison of clinical manifestations of actinic keratoses before and after treatment with 1% tirbanibulin ointment. The images show the presence of flakiness and irritation at the initial stage as well as the resolution after the end of the treatment.

We evaluated the presence of clinical signs and dermoscopic patterns of each treated AK within the area of tirbanibulin application. On day 57 of follow-up, 70% (n = 21) of the patients showed a complete clinical response (Figure 3, Figure 4 and Figure 5), whilst a partial clearance was observed in 9 cases (30%). Regardless of the resolution of the lesions, an improvement in skin texture was recorded in all the treated patients at day 57 of follow-up.

### 2.4. Molecular Docking Tirbanibulin

**Figure 6:**
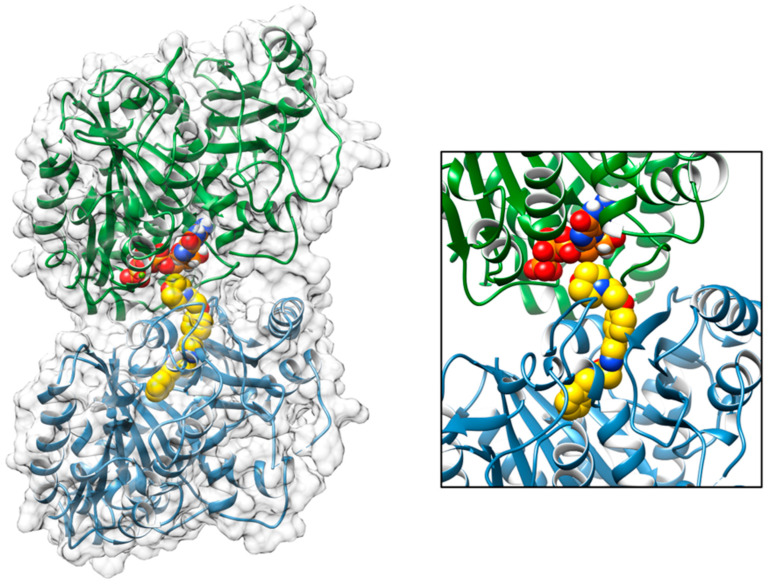
Molecular docking binding pose obtained between tirbanibulin and the α- and β-tubulin dimer. Tirbanibulin binding occurs at the colchicine-binding site of β-tubulin. The proteins are shown as cartoon with the α-tubulin in green and the β-tubulin in cyan, surrounded by a transparent grey surface. Tirbanibulin is shown as spheres, colored by atom type with carbon atoms in yellow. A GTP molecule, bound to the α-tubulin and interacting with the tirbanibulin, is shown as spheres, colored by atom type and with carbon atoms in orange. On the right, a closer view of the same binding pose is represented.

The interaction between the inhibitor tirbanibulin and the α- and β-tubulin dimer, a crystal structure of the complex, was obtained from the PDB database (PDBID: 6KNZ) [30] Sequence alignments showed that the bovine and human form of both the α- and β-tubulins had 100% sequence identity and structural alignment with the human inhibitor free form (PDBID: 7ZCW). Using Chimera software v 1.17.1, we were able to confirm that these proteins also share the same folding and an almost completely superimposable three-dimensional structure. Therefore, the crystallographic complex obtained between the bovine tubulins and the tirbanibulin (Figure 1) could also realistically indicate the binding configuration achieved by this compound in the human protein. The PDB crystallographic structure (PDBID: 6KNZ) showed that tirbanibulin binds at the colchicine-binding site of β-tubulin [32]. In order to evaluate the binding affinity between tirbanibulin and the β-tubulin pocket, a re-docking simulation was performed using the AutoDock Vina 1.2.3 software [33]. The best obtained binding configuration, superimposable with the crystallized ligand binding pose, showed that tirbanibulin had a predicted binding affinity of −9.7 kcal/mol with the α- and β-tubulin dimer (Figure 6).

### 2.5. Treatment Satisfaction

**Figure 7:**
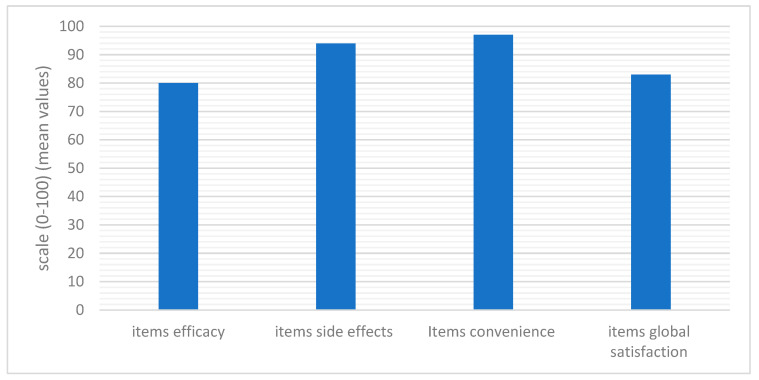
Evaluation of QoL in terms of treatment satisfaction: results of the different domains of the TSQM 1.4 on a scale from 0 to 100 evaluated at day 57 of follow-up.

Quality of life was assessed using the TSQM 1.4 questionnaire administered on day 57 of follow-up. The TSQM questionnaire consisted of 14 items assessing four domains: efficacy, side effects, convenience, and overall satisfaction. Patient satisfaction with medications, resulting from factors such as effectiveness, convenience (e.g., route of administration, dosing frequency), or drug side effects, is associated with better adherence and persistence to treatment. The TSQM showed high acceptability (100% of the participants answered all the items). Regarding medication adherence, the TSQM was able to discriminate satisfaction in the side effect and global satisfaction subscales. Considering the scores of the TSQM 1.4 ranged from 0 to 100 in each domain, we investigated different subscales. The higher scores were found in the convenience items (score = 97) and side effects items (score = 94). The other domains were also evaluated positively, with a score of 80 in effectiveness and a score of 83 in global satisfaction (Figure 7).

## 3. Materials and Methods

### 3.1. Enrolled Patients and Study Design

We conducted a single-center, prospective, real-life observational study. The aim of this study was to investigate in clinical practice the efficacy, safety, and quality of life (QoL) in patients with Aks treated with topical tirbanibulin 1% ointment.

### 3.2. Primary and Secondary Endpoints

In our study, tirbanibulin 1% ointment was applied topically to a 25 cm^2^ area on the scalp or face for 5 consecutive days. The inclusion criteria for participants were: (i) adult patients ≥ 18 years old; (ii) with 2–8 clinically typical AK lesions; and (iii) located on the face or scalp. The exclusion criteria included patients who were already undergoing topical treatment for the lesions. A single researcher performed the lesion assessments at the beginning, prior to the commencement of treatment, as well as before each subsequent follow-up appointment. On days 8, 15, 29, and 57, the AK lesions were assessed and compared to the day 1 baseline status, in accordance with phase 3 clinical trials. The primary endpoint was to evaluate the efficacy and safety of tirbanibulin. The secondary endpoint was to evaluate the treatment satisfaction with tirbanibulin.

### 3.3. Score Evaluation: Efficacy, Safety, and Treatment Satisfaction

The primary endpoint was the evaluation of efficacy assessed through clinical and dermoscopic monitoring of AKs. Clinical and dermatoscopic pictures of the lesions were obtained during the first visit and after each assessment visit. We described the presence or absence of classical dermoscopic features of AK for each patient: perifollicular vascular pseudo network, white/yellowish scales, and erythematous background with enlarged follicular openings. We considered Olsen’s classification system for grading AKs based on the overall thickness of each single lesion: grade 1 lesions are slightly palpable, grade 2 moderately thick, and grade 3 very thick and hyperkeratotic. This clinical classification system is commonly used in RCTs of AK therapies to define patient eligibility criteria, including mainly patients with AK grade1/2 and allows the exclusion of patients with hyperkeratotic lesions (i.e., Olsen grade 3) [34] The safety of the drug was assessed in terms of local skin reactions (LSRs). Six signs were evaluated: erythema, flaking or scaling, crusting, swelling, vesiculation or pustulation, and erosions or ulceration. A score on a 4-point scale was assigned to each observed sign with scores of 0 (absent), 1 (mild), 2 (moderate), and 3 (severe).

The secondary endpoint was to evaluate treatment satisfaction. This was assessed using the TSQM 1.4 within day 57 of follow-up. The TSQM (version 1.4) comprises 14 items across 4 domains focusing on effectiveness (3 items), side effects (5 items), convenience (3 items), and global satisfaction (3 items) of the medication over the previous 2–3 weeks, or since the patient’s last use. With the exception of item 4 (presence of side effects; yes or no), all items have 5 or 7 responses, scored from 1 (least satisfied) to 5 or 7 (most satisfied). Item scores are summed to give 4 domain scores, which are in turn transformed to a scale of 0–100 [35]. The “TSQM 1.4” is a questionnaire used to measure patient satisfaction with a specific pharmacological treatment in order to validate the patient’s perception of effectiveness, safety, and convenience, as well as any potential side effects. This questionnaire is very useful since it allows the clinician to evaluate adherence to the treatment.

### 3.4. Molecular Docking Simulation of the Interaction of Tirbanibulin

The crystal structure of the bovine α and β tubulin dimer associated with the inhibitor tirbanibulin was obtained from the PDB database (PDB ID: 6KNZ) [30]. This complex was selected because the bovine tubulin shows a 100% sequence identity compared to the human form. Sequence alignments were performed using the Protein BLAST (blast-p) suite (https://blast.ncbi.nlm.nih.gov/Blast.cgi?PAGE=Proteins, accessed on 7 February 2023) and the proteins Uniprot sequences (UNIPROT IDs: P81947, Q6B856, P68363, Q9BVA1). The inhibitor was re-docked into the binding site to evaluate the interaction energy. Molecular docking simulation was performed using the AutoDock Vina 1.2.3 program [32], whilst the prepare_receptor4.py and prepare_ligand4.py tools of the AutoDockTools4 program [18] were used to convert the receptors and drug structure files into pdbqt format. In this procedure, the receptor was treated as entirely rigid, and a simulation box of size x = 16.9 Å, y = 24.0 Å, z = 25.1 Å was placed around the crystallized ligand to select the binding pocket.

### 3.5. Statistical Analysis

Data were collected in a single database; descriptive and inferential statistical analyses were performed. The results were expressed as means or percentages, considering the type of each analyzed variable. We adopted the specific assessment time points as indicated in phase 3 randomized controlled clinical trial (RCTs) to monitor local side reactions (LSRs) of the drug in order to analyze the differences in terms of LSRs before and after treatment at different timepoints, i.e., baseline (BL) compared to day 8 (T8), day 15 (T15), day 29 (T29), and day 57 (T57). The parametric *t*-test or ANOVA was used for variable analyses. Results were considered statistically significant for *p*-values < 0.05, and SPSSv25 software was used.

## 4. Discussion

AKs, the most common precancerous lesions, arise on skin damaged by chronic exposure to UV light. AKs appear both as single lesions and or within the FC, which corresponds to extensively photodamaged perilesional tissue including non-visible subclinical lesions [5,14,15,16]. Although AKs may regress, they usually persist and can progress to SCC. For this reason, an early and effective treatment, capable of intervening on both clinical and subclinical lesions, is crucial to prevent progression to SCC [14,15,16]. Many topical treatments for AKs are often limited by poor tolerability and prolonged duration, with reduced patient adherence to therapy and compromised therapeutic success [3,17]. In 2020, the FDA approved the use of tirbanibulin in a 1% ointment formulation for the treatment of non-hyperkeratotic, non-hypertrophic Olsen grade I AKs of the scalp and face over a contiguous area of 25 cm^2^ with a daily application for 5 consecutive days [8,28]. Tirbanibulin has potent antitumor and antiproliferative activity due to its ability to reversibly bind to tubulin, inhibiting its polymerization and promoting microtubule rupture, thus inducing cell cycle arrest and apoptotic death of proliferating cells. Additionally, tirbanibulin can induce disruption of Src tyrosine kinase signaling, high levels of which play a role in the development of both AKs and SCC [28]. Unlike other treatments for AKs, tirbanibulin does not induce tissue necrosis with a low rate of severe LSRs, which favors good tolerability and a high safety profile of the drug. Two randomized, double-blind, parallel-group, vehicle-controlled, phase III clinical studies were conducted on 702 subjects with 4–8 AKs on the face or scalp within a contiguous area measuring 25 cm^2^ [8]. Efficacy was assessed with a primary endpoint of the proportion of patients with a complete reduction (100%) in the number of lesions on the application site at day 57 and a secondary endpoint of the proportion of patients with a partial reduction (≥75%) of the number of lesions, within the application area at day 57. In addition, the 1-year recurrence rate was evaluated. The pooled results from both studies demonstrated complete clearance in 49% of the patients (n = 174) and partial clearance in 72% of the patients (n = 255) treated with tirbanibulin [8]. Patients who achieved complete clearance were evaluated at one year and 47% of them relapsed (same location as baseline). Safety evaluations up to day 57 included recording of LSRs, appearance of pigmentation or scarring on the treated area, adverse events, ECG, physical examination laboratory parameters, and vital signs. Six signs of LSR were evaluated (erythema, desquamation, crusting, swelling, blisters, and erosions) and the severity was rated on a scale of 0–3 (0: none; 1: mild; 2: moderate; 3: severe). Mainly mild/moderate local skin reactions were reported. Erythema (93%) and desquamation (82%) were the most encountered reactions, as well as itching and pain at the application site. Local skin reactions peaked on day 8 of treatment and resolved spontaneously in approximately 2 weeks. In our real-life clinical experience with the use of tribanibulin 1% ointment on 30 patients, we obtained a better result than the phase 3 registration clinical trial (RCT). The effectiveness was assessed through clinical and dermoscopic objective examinations, conducted at baseline and compared to the final endpoint of our follow-up on day 57, in accordance with RCTs.

We reported complete resolution in 70% of the treated patients (n = 21), while partial resolution was observed in 30% of the population (n = 8). Regarding safety, our study did not document any new adverse events except for itching and burning sensations reported at the application site during the treatment cycle. The predominant LSR observed was erythema (83.3%), which closely aligns with the rate reported by Blauvelt et al. [8] (91%). The second most prevalent LSR was flaking or scaling (40%), which occurred less frequently than in the trials reported by Blauvelt et al. (82%) [8]. All local reactions had an average resolution within 10 days and resolved spontaneously without any lasting effects. No patient experienced systemic effects related to the drug. The LSRs did not cause any discomfort to patients, who all completed the treatment, thus confirming a favorable safety profile of tirbanibulin [36,37,38]. Furthermore, we assessed patient treatment satisfaction, resulting from factors such as effectiveness, convenience (e.g., route of administration, dosing frequency), or drug side effects. We observed adherence and persistence to the treatment. 

Most patients had previously received treatments using other topical approaches, such as 5′fluorouracil, diclofenac/HA, imiquimod, and other destructive therapies, molecules which have a longer duration of administration and a pro-inflammatory mechanism, which causes a moderate to severe local inflammatory response, resulting in reduced adherence to the therapy. The local use of tirbanibulin reported high satisfaction with the treatment modality based on its effectiveness. In fact, a short-duration treatment that was easy to use, not inducing any severe or locally disfiguring side effects contributed to a positive impact in the treated patients. This, in turn, helped treatment adherence with no patient discontinuing the study.

Tubulin inhibitors have frequently been explored in the context of cancer treatment, as they can disrupt cell division, resulting in the inhibition of cancer cell growth. Indeed, tubulin polymerization is a crucial process that forms microtubules, which are dynamic cytoskeletal structures essential for various cellular functions, including intracellular transport, cell division, and maintenance of cell shape. Moreover, the regulation of tubulin polymerization is a highly dynamic and tightly controlled process involving various cellular factors and signaling pathways. It plays a critical role in the maintenance of cell structure and function [32].

In this scenario, in order to provide a molecular explanation for tirbanibulin activity, we evaluated its binding to the tubulin dimer. From the docking result, we can suggest that the binding of this drug to the interface between α and β tubulin may interfere with its nucleation and elongation processes, thus favoring its intrinsic dynamic instability and leading to a shrinkage (i.e., depolymerization) of the microtubules. A RMSD of the value of 1.5Å was obtained by superimposing the structure bound to tirbanibulin with that determined in the absence of this ligand (PDB ID: 7ZCW). This confirms that the movements triggered by the drug binding could be responsible for an altered, or abolished, microtubule polymerization.

## 5. Conclusions

Recommendations for AKs offer a variety of treatment options for the different clinical presentations of AKs, but the driver for the right choice must be personalized, based on the number, location, and severity of the lesions, as well as on any patient comorbidities. 

It is still necessary to increase AK awareness among the general population and primary health care settings regarding the possible evolution of lesions into invasive tumours and the benefit of prevention in affected subjects most at risk.

Tirbanibulin 1% ointment represents an effective and innovative therapeutic option, with a dosage regimen that favours patient adherence, with an excellent safety and tolerability profile, related to a proapoptotic mechanism of action, which limits the inflammatory process at the site of application.

## 6. Future Directions

The use of tirbanibulin 1% ointment confirmed in our real-life study an excellent clinical outcome in AKs with an improvement of perilesional AKs photodamaged skin. Future studies will evaluate the utility of this molecule to treat large FC areas to better control subclinical AKs and help us achieve a more comprehensive assessment of the long-term effectiveness and safety of tirbanibulin in this chronic condition.

## 7. Limitation of the Study

The enrolment of a limited number of participants could be considered a limitation of our real life clinical trial. Additionally, the absence of a control group can make it challenging to establish a direct comparison between the treated group and an untreated one, thus compromising the ability to attribute the outcome specifically to the intervention being examined.

## Figures and Tables

**Table 1:** Patient characteristics of the study (*n* = 30).

Median Age (Years)	75 (60–93)
Gender	
Female	12 (40%)
Male	18 (60%)
Skin phototypes according Fitzpatrick classification	
I	12 (40%)
II	16 (53%)
III	2 (7%)
Prior therapies (26 patients)	
Curettage/cryosurgery	6 (24%)
Medical device Piroxicam 0.8% and sunfilters	7 (27%)
Diclofenac 3%	6 (24%)
5-Fluorouracil 5%	2 (8%)
5-Fluorouracil 0.5%+ salicylic acid 10%	2 (8%)
Imiquimod	4 (15%)
Laser-assisted photodynamic therapy	5 (19%)
5-Aminolevulinic acid patch	1 (4%)

**Table 2:** Systemic medications for associated comorbidities in total patients’ population (*n* = 26).

Acetylsalicylic Acid	20.75%
Thiazide Diuretics	11.30%
Angiotensin II Receptor Blockers	11.30%
Statins	11.30%
Antiplatelet Agents	9.43%
Metformin	7.54%
Levothyroxine	7.54%
Potassium-Sparing Diuretics	5.66%
Beta-Blockers	3.77%
Calcium Channel Blockers	3.77%
Incretin	1.88%
Insulin	1.88%
Carbamazepine	1.88%

## Data Availability

All data generated or analyzed during this study are included in this article. Further enquiries can be directed to the corresponding author.
